# Heightened Olfactory Sensitivity in Young Females with Recent-Onset Anorexia Nervosa and Recovered Individuals

**DOI:** 10.1371/journal.pone.0169183

**Published:** 2017-01-06

**Authors:** Mette Bentz, Johanne Guldberg, Signe Vangkilde, Tine Pedersen, Kerstin Jessica Plessen, Jens Richardt Moellegaard Jepsen

**Affiliations:** 1 Child and Adolescent Mental Health Centre, Mental Health Services in the Capital Region of Denmark, Copenhagen, Denmark; 2 Department of Clinical Medicine, Faculty of Health and Medical Sciences, University of Copenhagen, Copenhagen, Denmark; 3 Department of Psychology, Faculty of Social Sciences, University of Copenhagen, Copenhagen, Denmark; 4 Danish Research Centre for Magnetic Resonance, Centre for Functional and Diagnostic Imaging and Research, Copenhagen University Hospital Hvidovre, Hvidovre, Denmark; 5 Lundbeck Foundation Center for Clinical Intervention and Neuropsychiatric Schizophrenia Research (CINS) and Center for Neuropsychiatric Schizophrenia Research (CNSR), Psychiatric Center Glostrup, Glostrup, Denmark; Universite de Bretagne Occidentale, FRANCE

## Abstract

**Introduction:**

Olfaction may be related to food restriction and weight loss. However, reports regarding olfactory function in individuals with anorexia nervosa (AN) have been inconclusive.

**Objective:**

Characterize olfactory sensitivity and identification in female adolescents and young adults with first-episode AN and young females recovered from AN.

**Methods:**

We used the Sniffin’ Sticks Odor Threshold Test and Odor Identification Test to assess 43 participants with first-episode AN, 27 recovered participants, and 39 control participants. Participants completed the Importance of Olfaction questionnaire, the Beck Youth Inventory and the Eating Disorder Inventory. We also conducted a psychiatric diagnostic interview and the Autism Diagnostic Observation Schedule with participants.

**Results:**

Both clinical groups showed heightened olfactory sensitivity. After excluding participants with depression, participants with first-episode AN identified more odors than recovered participants.

**Conclusion:**

Heightened olfactory sensitivity in AN may be independent of clinical status, whereas only individuals with current AN and without depression show more accurate odor identification.

## Introduction

Anorexia nervosa (AN) is characterized by egosyntonic food restriction, a disturbed body perception and a persistent pursuit of thinness, which may lead to a state of severe underweight.[1] AN most often emerges in adolescence and occurs more frequently in girls than in boys.[2]

Olfactory abilities increase with age until approximately 20 years.[3,4] Females tend to display olfaction abilities that are superior to males.[5] States of hunger and satiety modulate olfaction in healthy individuals,[6] and pleasant odors activate the reward system of the brain.[7] Studies have suggested that individuals with AN have altered reward processing of illness-related stimuli including food, and these alterations may also include odors.[7–9] The possible role of olfaction in food restriction has motivated studies of olfactory characteristics in individuals with AN. Moreover, evidence of altered olfaction in a number of psychiatric disorders, e.g. depression, anxiety, and schizophrenia, has identified olfaction as a possible avenue for gaining further insight into the pathophysiology of these disorders.[10,11]

Olfactory function involves peripheral, as well as central processes.[12] The peripheral processes include odor sensitivity and occur primarily in the olfactory receptors of the nasal epithelium and olfactory bulb. The central processes include odor identification and involve the primary olfactory cortex in the temporal lobes, higher order brain processes such as reward processing in the orbitofrontal cortex, attention and memory.[8,12–14]

Few studies have assessed olfaction in AN, seven of which included adolescents. However, only three studies reported results separately for this age group.[15–17] One of these three studies in adolescents observed lower odor sensitivity (higher threshold) and normal odor identification,[15] whereas another study observed normal sensitivity and more precise identification in adolescents with AN.[16] The third and largest study with adolescents observed higher sensitivity in participants with AN.[17] The largest reported study included 64 adults with AN and observed higher odor sensitivity, but normal odor identification compared with controls.[18] Interestingly, participants with AN showed an association between olfactory sensitivity and illness-related factors related to positive outcome, e.g. higher BMI and less body dissatisfaction were associated with superior sensitivity.

Neither odor sensitivity nor identification have been investigated in individuals who have fully recovered from AN. However, two longitudinal studies observed improvement of a reduced sensitivity after short-term weight gain in adolescents only[15] and in a combined sample of adolescents and adults with AN,[19] whereas another study reported no change in adolescents with AN.[17] Additionally, a longitudinal study observed improvement in overall olfaction in adults with chronic AN after weight gain.[20]

Depression and anxiety are associated with reduced and increased olfaction, respectively.[21,22] Thus, depression and anxiety may influence findings in individuals affected by AN due to their common co-occurrence.[23] Indeed, a study of adolescents with AN observed increased identification ability only when participants with psychiatric comorbidity, mainly depression, were omitted from analysis.[16]

Olfaction plays an important, but often not consciously perceived, role in human social interaction.[24] Odors may enhance detection of fear in others, and odors from well-known others may reduce stress in disturbing situations.[24] Individuals with schizophrenia exhibit an association between impaired odor identification and social dysfunction.[25,26] A review also confirmed impaired odor identification in individuals with autism spectrum disorder, a disorder characterized by impairment of social function.[27] Although subgroups of individuals with AN repeatedly have shown impaired social function,[28–30] potential associations with aspects of olfaction in AN remain unexplored.

A recent review of olfaction in AN concluded that findings are contradictory regarding several aspects of olfaction.[11] Furthermore, the authors note that comparisons between studies have been hampered by differences in methodology, age and sex of participants, duration of illness, and control of other confounders.[11] Specifically, few studies controlled for the effects of depression, duration of illness and age.

Olfactory impairments are associated with lower quality of life in individuals with olfactory dysfunction.[31,32] Healthy subjects vary greatly in the relevance they ascribe to olfaction,[33] but this has not yet been studied in individuals with AN. However, in two studies underweight and weight-recovered individuals with AN reported higher sensitivity to a broad array of sensory stimuli than control participants,[34,35] and the self-reported sensory sensitivity was associated with difficulties in emotional regulation.[34] Finally, underweight individuals, as well as those weight-recovered from AN indicated avoidance toward sensory experiences, and higher levels of perceived sensitivity, both of which were associated with a higher body dissatisfaction.[35] Such findings link the subjective experiences of bodily senses, including smell, to a core feature of AN, namely body dissatisfaction.

The primary aim of the current study was thus to characterize olfactory sensitivity and identification of odors in female adolescents and young adults either with first-episode AN or fully recovered from AN while taking into account duration of illness, comorbid disorders, and illness-related factors. Exploratively, we further aimed to assess the attributed importance of olfaction in these two groups. Additionally, we aimed to examine whether a potential difference between groups in olfactory sensitivity and identification was associated with relevant clinical features such as symptoms of depression or anxiety in any of the groups, or with AN-specific features in the first-episode AN group. Finally, we aimed to assess a possible association between olfaction and social function within each diagnostic group.

We hypothesized that participants with first-episode AN and recovered from AN would display increased olfactory sensitivity and more precise odor identification than control participants. In the face of inconsistent earlier findings, we based this hypothesis on the two largest prior studies in adolescents with AN on olfactory sensitivity[17] and identification.[16]

## Methods

### Participants

We consecutively invited 14–17 yr old female patients with AN at the Child and Adolescent Mental Health Center (CAMHS) between July 2012 and March 2015, unless participation was contraindicated due to their physical condition. In addition, we recruited three 18–21 yr old patients with AN from the Stolpegaard Psychotherapy Center. Both centers are part of Mental Health Services in the Capital Region of Denmark, where treatment is covered by state insurance and initiated soon after referral. The inclusion criteria for participants with first-episode AN included a recent onset (within a maximum of 12 months), first-episode of AN (ICD-10: F50.0 or F50.1)[1] and a BMI ≤ 18.5 kg/m^2^ for participants 16 years or older or a BMI-percentile corrected for age ≤ 25^th^ percentile for 14–15 yr old participants.[36] Prior psychological treatment of the current episode was allowed, but prior episodes of AN were not.

Eligible participants recovered from AN were identified through a quality assurance survey.[37] Inclusion criteria included recovery from AN (ICD-10: F50.0 or F50.1),[1] a normal body weight for a minimum of one year prior to inclusion, eating and weight concerns within normal range [defined as a global score of the Eating Disorders Examination (EDE) within one standard deviation (SD) of the published mean],[38] no eating disorder pathology as assessed by EDE diagnostic questions, no ongoing treatment for an eating disorder, and a generally favorable outcome [≥ nine points on the Morgan Russell Outcome Assessment Schedule (MROAS)].[39] We assessed severity of prior AN (at onset) in the participants recovered from AN using clinical data recorded when they were admitted to treatment. The cohort participating in the present study was assessed in a prior study investigating social functioning.[30]

We invited control participants via notice board advertisements for a study concerning social functioning. Advertisements were distributed in colleges, state schools and halls of residency in the catchment area of the hospital. Inclusion criteria for controls included a normal body weight throughout lifetime and no history of mental illness (Axis I disorders), [40] (minor exceptions included transient childhood tics and adjustment disorders).

Exclusion criteria for all three groups included infantile autism (F84.0) or Asperger´s syndrome (F84.5). We also excluded participants with first-episode AN and controls that currently were treated with psychotropic drugs, due to their potential influence on olfaction, e.g. selective serotonin reuptake inhibitors (SSRI).[15] This did not compromise the representativeness of individuals with first-episode AN, because we recruited participants early in their treatment, when psychotherapeutic intervention usually is the first choice of treatment. Evidence suggests that individuals recovered from AN often have psychiatric disorders such as anxiety and depression.[23,41] Therefore, we did not exclude recovered individuals with past or present psychopharmacological treatment.

Participants or legal caretakers gave written and informed consent according to the guidelines of the Danish Health Authority. The Danish Data Protection Agency and The Regional Scientific Ethical Committee in the Capital Region of Denmark (project number H-2-2012-027) approved this study.

Our final sample included 43 females with first-episode AN, 28 females recovered from AN, and 41 control participants (Table 1).[42]

**Table 1:** Demographic and clinical characterization of participants with first-episode AN, participants recovered from AN, and controls.

				**Test statistics**		**Effect size**^a^ **(p)**		
	FeAN (N = 43)	RecAN (N = 28)	Con (N = 41)		p	FeAN vs. RecAN	FeAN vs. Con	RecAN vs. Con
**1.a AT THE TIME OF STUDY PARTICIPATION**								
**Age, mean (SD) range**	16.1 (1.5) 14.1–21.5	18.4 (1.6) 15.7–21.6	17.7 (2.2) 14.0–22.5	F(2,109): 16.119	< .001	-1.48	-0.85	0.36
						(< .001)	(< .001)	(ns)
**Parents´ highest education, years (SD)**	16.1 (2.3)	14.8 (2.8)	15.3 (2.4)	F(2,109): 2.591	.08			
**Living with both parents, N (%)**	24 (56%)	17 (61%)	21 (51%)	Chi^2^(2) = .613	.74			
**BMI (kg/m^2^), mean (SD)**	16.6 (1.2)	21.3 (1.8)	22.0 (2.6)	F(2,109): 92.608	< .001	-3.07	-2.67	-0.31
						(< .001)	(< .001)	(ns)
**BMI percentile corrected for age^b^, mean (SD)**	7.5 (7.6)	47.3 (19.0)	56.9 (21.2)	F(2,109): 101.948	< .001	-2.75	-3.10	-0.48
						(< .001)	(< .001)	(ns)
**Comorbid depressive disorder^c^^,^^d^, N (%)**	8 (19)	3 (11)		Fishers exact (2-sided)	.51			
**Comorbid OCD, N (%)**	3 (7)	0 (0)		Fishers exact (2-sided)	.27			
**Comorbid anxiety other than OCD, N (%)**	4(9)	5 (18)		Fishers exact (2-sided)	.30			
**EDE global score, mean (SD)**	2.8 (1.5)	0.6 (0.5)		t(69): 9.119	< .001	1.97		
**RecAN duration of treatment (months)^e^, (SD)**		23.9 (11.8)						
**RIAS Intelligence Quotient, mean (SD)**	107.7 (10.5)	102.8 (11.5)	107.3 (9.5)	F(2,109): 2.137	.12			
**EDI^f^ Eating Disorder Risk Composite, mean (SD)**	47.7 (10.1)	36.5 (6.4)	36.1 (7.0)	F(2,105): 24.861	< .001	1.32	1.33	0.06
						(< .001)	(< .001)	(ns)
**EDI Body Dissatisfaction Scale T-score, mean (SD)**	49.0 (8.8)	37.9 (8.2)	36.9 (7.7)	F(2,105): 25.254	< .001	1.31	1.46	0.13
						(< .001)	(< .001)	(ns)
**EDI Interoceptive Deficits Scale T-score, mean (SD)**	49.4 (8.1)	35.3 (8.4)	36.0 (8.2)	F(2,105): 34.794	< .001	1.71	1.64	-0.08
						(.001)	(< .001)	(ns)
**BYI Anxiety Index T-score^h^, mean (SD)**	57.1 (9.8)	51.8 (12.7)	47.8 (9.9)	F(2,109): 8.092	.001	0.47	0.94	0.35
						(ns)	(< .001)	(ns)
**BYI Depression Index T-score^h^, mean (SD)**	60.9 (10.8)	49.8 (12.3)	48.5 (9.5)	F(2,109): 16.262	< .001	0.96	1.22	0.12
						(.001)	(< .001)	(ns)
**ADOS-Total**	2.77 (3.1)	2.79 (3.2)	1.05 (1.5)	F(2,109): 5.450	.006	-0.06	0.71	0.70
						(ns)	(.005)	(.03)
**1.b AT THE TIME OF TREATMENT INITIATION**								
**Age, mean (SD)**	16.1 (1.5)	14.8 (1.6)		t(69) =	.001	0.84		
				3.357				
**BMI percentile corrected for age, mean (SD)**	3.8 (5.4)	4.8 (6.0)		t(69) =	.5			
				- .722				
**EDE global score at time of treatment^g^, mean (SD)**	2.8 (1.5)	2.8 (1.3)		t(54) =	1.0			
				- .051				
**Binge-purge subtype AN, N (%)**	4 (9%)	4 (14%)		Fishers exact (2-sided)	.7			
**Comorbid depressive disorder, N (%)**	8 (19%)	4 (14%)		Fishers exact (2-sided)	.8			

FeAN = first-epsiode AN participants; RecAN = recovered AN participants; Controls = control participants; ns = non-significant (p< .05); BMI = Body Mass Index; EDI = Eating Disorder Inventory-3; OCD = obsessive-compulsive disorder; EDE = Eating Disorder Examination; RIAS = Reynolds Intellectual Assessment Scales; ADOS-Total = Autism Diagnostic Observation Schedule-2, Communication and Social Interaction Total algorithm score.

a. Between-group effect sizes presented as Cohen´s d.

b. BMI percentiles corrected for age <0.02 are calculated as = 0.02. Participants>20 yr old were given percentile of 20 yr olds.

c. Depressive disorder includes mild depression, moderate depression and severe depression according to ICD-10.

d. We used the term “comorbid” although anxiety and depression are only comorbid to AN in the case of the participants with first-episode AN.

e. We use months in treatment for the recovered as a proxy for duration of AN, knowing that this might not be entirely precise, because AN typically has a gradual onset.

f. EDI-3 answers are missing from four FeAN.

g. EDE data available from time of treatment for recovered participants, N = 13 (46%).

h. T-scores are derived from American norms in the absence of Danish norms for adolescents.

The three groups did not differ with respect to parental education, number of participants living with both parents, or intelligence level. However, participants with first-episode AN were younger than the two other groups (Table 1). The participants with first-episode AN and those recovered did not differ concerning their rate of current comorbid psychiatric disorders as assessed with the Schedule for Affective Disorders and Schizophrenia in School-Age Children—Present and Lifetime version (K-SADS-PL) (Table 1).[43] The subgroup of participants recovered from AN, who had other psychiatric disorders (N = 8), were all in remission, and these disorders were assessed not to interfere with a generally favorable outcome (MROAS),[39] yet three recovered participants used an SSRI at the time of testing. Clinical characteristics did not differ between the two diagnostic groups when they started treatment (Table 1), apart from the fact that recovered participants were younger at admission (Table 1). We omitted participants with anosmia from analyses of olfactory sensitivity and identification (recovered: N = 1, controls: N = 2). Anosmia was defined as a score of zero on the Odor Threshold Test, or ≤ 7 correct answers on the Odor Identification Test, as the latter is randomly obtainable.[44]

### Measures

We used the Sniffin’ Sticks Odor Threshold Test and Odor Identification Test[45] to assess olfactory performance. The Sniffin’ Sticks battery is the test most frequently used in studies of individuals with AN.[11] Both tests consist of felt-tip pens with odorants and were administered to both nostrils simultaneously. The Odor Threshold Test contains *n*-buthanol in 16 dilutions of increasing strength. They were presented in a triple forced-choice paradigm in which one contains the target odor (*n*-buthanol) and the other two contain a non-odorant solvent. The final score was calculated as the mean of the last four turning points, i.e. either the highest dilution in which the participant correctly identified the odorant twice in a row, or the lowest (more concentrated) dilution in which the participant failed to identify the dilution once. Higher scores thus represent higher sensitivity. The Odor Identification Test consists of 16 different common odors, each with a forced-choice answer with four alternatives. Scores represent the number of odors correctly identified.

We differed from the recommended procedure of the test in three ways. [45] First, we did not blindfold our participants, but let them face away and covered the color code of the sticks to allow for a more comfortable administration of the threshold test. Second, we only required participants to identify one stick correctly instead of two in a row before moving up the staircase in order to increase tolerability and reduce administration time of the test. Third, we presented the sticks in a pseudo-random order instead of the predetermined sequence to prevent participants from guessing based on the sequence of presentation.

We assessed participants’ subjective experience of olfaction with the ‘Importance of Olfaction’ questionnaire.[33] The questionnaire consists of three subscales with six items each, formulated as personal statements on a Likert Scale, with higher scores representing more importance ascribed to olfaction. The *Association Subscale* assesses emotions, memories, and evaluations triggered by the sense of smell. The *Application Subscale* assesses the degree to which a person uses his or her sense of smell in daily life. The *Consequence Subscale* assesses the conclusions someone draws from their olfactory impressions. The questionnaire includes five items with food-related content, which may potentially bias the results in participants with first-episode AN due to prevalent food aversion.[46] Additionally, it includes two questions pertaining to response bias when presenting for treatment of olfactory loss. They were not considered relevant for the present study and thus were not included in the analyses. The original questionnaire was translated into Danish by one of the authors and back-translated into German by a Danish-speaking German and then approved by the author.

Participants and parents were interviewed using a K-SADS-PL interview, and participants completed the Beck Youth Inventory (BYI)[47] that includes dimensional measures of depression symptoms (BDI-Y) and anxiety (BAI-Y). Preliminary analysis revealed a strong bivariate correlation between the BDI-Y and BAI-Y scores (*r*^2^ = .80, p< .001 in the total sample). Moreover, the factor structure of the BYI indicate that the two scales tap into the same domain of emotional distress.[48] However, depressive disorders reduce the ability of olfaction,[21] whereas the presence of anxiety enhances olfaction,[22] which is why we analyzed both scales. Participants also completed the Eating Disorder Inventory, third edition (EDI-3),[49] to describe thoughts and emotions related to AN.

We used the Communication and Social Interaction Total algorithm of the Autism Diagnostic Observation Schedule (ADOS-Total), second edition, module 4, Danish version to assess social function.[50] All ADOS observations were performed by the same researcher, who met the standard requirement for research reliability of ADOS administration, while assessing interrater reliability against an expert in a random sample of cases based on video recordings.[42] Finally, we assessed intelligence level with the Reynolds Intellectual Assessment Scales (RIAS).[51]

The assessment procedures lasted approximately 8 hours, distributed over 1–3 visits, preferably scheduled in the afternoons after school hours. All tests, clinical interviews, and questionnaires in the study were administered in a fixed, pseudo-randomized order, taking variety into account, and beginning with the ADOS interview. Tests of olfaction were administered in the middle of the assessment procedures. Participants were offered breaks when needed.

### Statistical procedures

#### Main analyses

Statistical analyses were carried out in IBM SPSS statistics version 22.[52] Odor Threshold Test scores and Odor Identification Test scores were analyzed separately with one-way analyses of variance (ANOVAs) after checking for model assumptions. We used a Bonferroni-adjusted level of significance of 2.5% (p< .025) for the two main analyses of olfaction tests scores to control for type I errors when testing two hypotheses. Post hoc analyses of difference between groups are reported as Cohen´s d (pooled standardized mean difference).

To assess potential impact of factors known to be associated with olfaction we repeated the ANOVAs of the two olfaction test scores four times, each time excluding the participants with the characteristic in question: smoking on a daily basis (N = 15; 3 first-episode AN, 5 recovered, 7 controls),[53] current major depressive disorder (N = 11; 8 first-episode AN, 3 recovered),^37^ anxiety disorder other than OCD (N = 9; 4 first-episode AN, 5 recovered),[22] serotonergic medication (N = 3 recovered).[15] If these sensitivity analyses did not change the main results, then the subgroups were kept in the main analysis to maintain statistical power.

We adjusted the outcome scores of the olfaction test and questionnaire by age when weighting cases with a standardized frequency weight[54] based on four age groups (14–16.5 yr, 16.5–17.25 yr, 17.25–18.5 yr, 18.5–22.5 yr) due to the between-group differences in mean age (Table 1), i.e. to prevent potential effects of the significant between-group mean age difference.

Before performing the weighting procedure we inspected scatterplots of each variable with age and observed no outliers. For the additional sensitivity analyses, age weighting was based on three age groups to verify that no age group was smaller than N = 3.

#### Exploratory analyses

To assess group differences in the mean scores of the three subscales of the Importance of Olfaction questionnaire (Association, Application, and Consequence), we conducted a one-way multivariate analysis of variance (MANOVA) after checking for model assumptions. In the case of a significant result, we performed post hoc ANOVAs. The outliers of the age-weighted subscale scores (N = 3) were truncated to the next observed age-weighted subscale score in that particular group. As a sensitivity analysis, we performed an ANOVA with the Importance of Olfaction total scores excluding all questionnaire items with food-related content. One item from the Association subscale, four items from the Application subscale and no items from the Consequence subscale had food-related content and thus were removed.

We performed two sets of ANCOVAs with appropriate checking for model assumptions; one set of ANCOVAs with age-weighted Odor Threshold Test score, and one with age-weighted Odor Identification Test score as the dependent variable. The purpose of the ANCOVAs was to a) assess whether a potential difference between groups in olfactory sensitivity or identification was associated with severity of depressive or anxiety symptoms, and b) to assess whether olfaction was associated with social functioning in the groups. Each ANCOVA included group as fixed factor and either BDI-Y, BAI-Y, or ADOS-Total score as independent variable besides group. Interaction terms between group and each of the remaining independent variables were tested and included if significant.

For the analysis of potential associations between scores of each of the olfaction tests (Odor Threshold Test and Odor Identification Test) and the clinical variables, we performed multiple regression analyses within the group of participants with first-episode AN. BMI adjusted for age and the EDI-3 Body Dissatisfaction Scale score were independent variables, and each of the olfaction measures were dependent variables. We employed no correction of the level of significance in the exploratory analyses.

## Results

### Olfactory sensitivity

The between-group difference on the mean Odor Sensitivity Test scores was significant with a medium effect size (Table 2).

**Table 2:** Sniffin´Sticks Odor Identification Test and Odor Threshold Test results.

Measure	Participants, Mean (SD)			ANOVA			Pairwise comparisons: Effect sizes d (p)^a^		
	FeAN	RecAN	Con	F	p	R^2^	FeAN vs. RecAN	FeAN vs. Con	RecAN vs. Con
**Odor Threshold Test**^b^	N = 43 9.18 (1.52)	N = 27 8.85 (2.13)	N = 397.45 (2.31)	F(2,106): 8.364	< .001	.14	0.18 (ns)	0.88 (.001)	0.63 (.04)
**Odor Threshold Test excluding smokers**	N = 40 9.33 (1.48)	N = 22 8.86 (2.09)	N = 327.50 (2.39)	F(2,91): 7.864	.001	.15	0.26 (ns)	0.92 (.001)	0.61 (ns)
**Odor Threshold Test excluding participants with depression**	N = 35 8.94 (1.50)	N = 24 9.01 (2.07)	N = 397.37 (2.27)	F(2,95): 7.696	.001	.14	-0.04 (ns)	0.82 (.003)	0.75 (.005)
**Odor Threshold Test excluding participants with anxiety**^d^	N = 39 8.96 (1.44)	N = 22 8.95 (1.99)	N = 397.38 (2.29)	F(2,97): 7.971	.001	.14	0.01 (ns)	0.83 (.001)	0.73 (.02)
**Odor Threshold Test excluding participants using SSRI**	N = 43 9.12 (1.50)	N = 24 8.89 (2.20)	N = 397.37 (2.28)	F(2,103): 8.827	< .001	.15	0.12 (ns)	0.93	0.70 (.01)
								(< .001)	
**Odor Identification Test**^c^	N = 43 12.98 (1.24)	N = 27 11.96 (1.64)	N = 3912.65 (1.83)	F(2,106): 3.581	.03	.06	0.70 (.03)	0.21 (ns)	-0.40 (ns)
**Odor Identification Test excluding smokers**	N = 40 12.86 (1.29)	N = 22 11.80 (1.64)	N = 3212.62 (1.94)	F(2, 91): 3.177	.05	.07	0.72 (.04)	0.14 (ns)	-0.46 (ns)
**Odor Identification Test excluding participants with depression**	N = 35 12.92 (1.20)	N = 24 11.77 (1.62)	N = 3912.66 (1.84)	F(2, 95): 4.007	.02	.08	0.81 (.02)	0.17 (ns)	-0.51 (ns)
**Odor Identification Test excluding participants with anxiety**^d^	N = 39 12.95 (1.14)	N = 22 11.87 (1.69)	N = 3912.66 (1.82)	F(2, 97): 3.421	.04	.07	0.75 (.03)	0.19 (ns)	-0.45 (ns)
**Odor Identification Test excluding SSRI**	N = 43 12.85 (1.24)	N = 24 11.80 (1.64)	N = 3912.66 (1.83)	F(2, 103): 3.578	.03	.07	0.72 (.03)	0.12 (ns)	-0.49 (ns)

SD = standard deviation; d = Cohen’s d; FeAN = participants with first-episode AN; RecAN = participants recovered from AN; Controls = control participants; ns = non-significant.

a. We used Tukey´s or Games-Howell test for post hoc comparisons depending on the homogeneity of variances.

b. Odor Threshold Test scores represent mean of last 4 turning points.

c. Odor Identification Test scores represent mean number of odors correctly identified.

d. Anxiety disorders other than OCD.

Post hoc comparisons revealed that participants with first-episode AN and recovered participants did not differ significantly from each other and had significantly higher mean Odor Sensitivity Test scores than controls.

When excluding participants who smoked, had a diagnosis of depression or anxiety, or were currently treated with SSRI medication, results of the ANOVA were unaltered (Table 2). Post hoc comparison results stayed the same, except when participants who smoked were excluded. In this case only the mean Odor Sensitivity Test score of individuals with first-episode AN was significantly higher than the score of the controls.

### Odor identification

Participants in the three groups did not significantly differ in their ability to identify odors within the Odor Identification Test (Table 2). However, when excluding participants with a comorbid diagnosis of depression, the ANOVA revealed a significant between-group difference. Post hoc tests showed that the remaining participants with first-episode AN identified significantly more odors than the remaining recovered participants (Table 2). The ANOVAs without participants who either smoke, had a diagnosis of anxiety, or were currently treated with SSRI medication showed no statistically significant difference between groups.

### Exploratory results

#### Subjective Importance of Olfaction

The MANOVA revealed a significant between-group difference in the Importance of Olfaction questionnaire subscale means with a small effect size [F(6, 214): 3.069, p = .01; Wilk´s Lambda = .848; R^2^ = .079]. Post hoc ANOVAs showed that only the Association subscale was significantly different between the groups, because participants with first-episode AN indicated a significantly lower importance of olfaction than recovered participants and controls (Table 3).

**Table 3:** Importance of Olfaction Questionnaire results, age weighted mean.

	Participants, Mean (SD)			ANOVA			Pairwise comparisons: Effect size d^a^ (p)^b^		
Measure	FeAN	RecAN	Controls	F(2,109)	p	R^2^	FeAN vs. RecAN	FeAN vs. Con	RecAN vs. Con
**Questionnaire total score**	25.32 (7.87)	29.70 (5.98)	29.76 (7.38)	F(2,109): 4.941	.01	.08	-0.63 (.04)	-0.58 (.02)	0.001 (ns)
**Association subscale**	8.38 (3.38)	10.32 (2.64)	10.59 (2.71)	F(2,109): 6.672	.002	.11	-0.64 (.02)	-0.72 (.003)	-0.10 (ns)
**Application subscale**	8.34 (3.28)	9.22 (3.34)	10.10 (3.32)	F(2,109): 2.989	.05	.05	-0.27 (ns)	-0.53 (.04)	-0.26 (ns)
**Consequence subscale**	8.58 (3.06)	10.16 (2.19)	9.20 (2.86)	F(2,109): 2.735	.07	.05	-0.59 (.06)	-0.21 (ns)	0.38 (ns)
**Questionnaire total score excluding 5 food-related items**	18.39 (6.53)	21.28 (4.81)	20.45 (5.82)	F(2,109): 2.369	.10	.04	-0.50 (ns)	-0.33 (ns)	0.16 (ns)

SD = standard deviation; FeAN = participants with first-episode AN; RecAN = participants recovered from AN; Controls = control participants; ns = non-significant.

a. d = Cohen’s d.

b. b. We used Tukey´s or Games-Howell test for post hoc comparisons depending on the homogeneity of variances.

After exclusion of the food-related questions, the ANOVA showed no significant difference between groups (Table 3).

#### Associations between olfaction and clinical variables

Adjusting the between-group comparison of mean Odor Threshold Test scores for the severity of depressive (BDI-Y) or anxiety symptoms (BAI-Y) did not change the pattern of results observed in the unadjusted analysis (Table 4).

**Table 4:** Unadjusted and adjusted olfaction test score means with confidence intervals.

		Unadjusted		Adjusted for depression^a^		Adjusted for anxiety^b^	
	N	mean	95% CI	mean	95% CI	mean	95% CI
**Olfactory threshold**							
FeAN	43	9.18*	8.56–9.78	9.32*	8.68–9.96	9.17*	8.55–9.79
RecAN	27	8.85*	8.09–9.61	8.77*	8.01–9.54	8.85*	8.09–9.61
Controls	39	7.45	6.82–8.08	7.35	6.70–8.00	7.47	6.81–8.13
**Odor identification**							
FeAN	43	12.98	12.51–13.46	13.02	12.51–13.53	12.96	12.47–13.45
RecAN	27	11.96	11.36–12.55	11.93	11.32–12.54	11.95	11.35–12.55
Controls	39	12.65	12.15–13.14	12.62	12.10–13.13	12.67	12.15–13.19

FeAN = participants with first-episode AN; RecAN = participants recovered from AN; Controls = control participants

* = Statistically different from controls (Bonferroni-adjusted p< .05).

a. Depressive symptoms measured by Beck Depression Inventory-Youth Index, evaluated at the value of T-score = 53.44.

b. Anxiety symptoms measured by Beck Anxiety Inventory-Youth Index, evaluated at the value of T-score = 52.76.

Likewise, adjusting the between-group comparison of mean Odor Identification Test scores for BDI-Y or BAI-Y did not change the pattern of results observed in the unadjusted analysis (Table 4).

In the regression analysis within the group of first-episode AN participants, neither EDI-3 Body Dissatisfaction Scale scores (p = .43) nor BMI-percentile adjusted for age (p = .25) significantly predicted Odor Threshold Test scores. In the second regression analysis, lower EDI-3 Body Dissatisfaction Scale scores, representing less severe body image disturbance, significantly predicted higher Odor Identification Test scores with a small effect size, [β = —.37, p = .02, R^2^ = .136], whereas BMI-percentile adjusted for age did not (p = .54).

#### Associations between olfaction and social functioning

The ADOS-Total score was not a significant predictor of olfactory sensitivity [F(1,105) = 0.728, p = .40, R^2^ = .01] nor of odor identification [F(1,105) = 2.499, p = .12, R^2^ = .02]. Further, the pattern of group differences in olfactory performance did not change, when ADOS-Total was added as a predictor.

## Discussion

In terms of the primary aims of the present study, individuals with first-episode AN and recovered individuals demonstrated higher olfactory sensitivity than controls and did not differ from one another. Furthermore, the three groups did not differ in their ability to identify odors. However, after excluding participants with a comorbid depressive disorder, the remaining participants with first-episode AN were able to identify odors more accurately than recovered participants. Thus, our finding supports earlier findings of superior olfactory functions in persons with AN, specifically concerning young persons with a short duration of illness.

We observed no associations between the olfactory functions and social functioning. We cannot discern whether this lack of association reflects truly unrelated areas of functioning in individuals with AN, or whether it reflects the limited range of scores among our participants, because the impairment of social function in our groups without ASD may be less severe than in other diagnostic groups.

Olfaction depends on the orbitofrontal cortex (OFC), an area also involved in assigning reward value to stimuli.[55,56] Alterations in the activation of the OFC in response to food in individuals with AN are documented,[8] a process involved in aberrant learning of aversion toward food.[57,58] Hence, superior olfaction and especially superior olfactory sensitivity might enhance this aversive learning process, thereby exacerbating AN symptoms.

Prior studies reported that individuals with AN showed either reduced,[15,19,59] normal,[16,20,60–62] or superior[17,18,21] olfactory sensitivity. Several factors, such as different test used, small and heterogeneous samples, lack of control for medication, duration of AN, or the presence of comorbid disorders may explain this inconsistency as outlined in a recent review.[11] With regard to duration of AN, we recruited individuals with first-episode AN shortly after they presented for treatment for the first time, thus minimizing effects of chronic starvation, which may affect olfaction differentially over time. Fasting for 24 hours increases olfaction in healthy individuals,[63] but long-lasting starvation may decrease cell renewal of the olfactory epithelium possibly resulting in reduced sensitivity.[17,19,21] It has been noted, that the sensitivity scores of the controls were unusually low in a prior study, which in part may explain findings of high sensitivity in the clinical group.[11] In comparison, the mean threshold score of the controls in our study falls between the published mean for 5-15-year-old healthy females and that of 16-36-year-old healthy females. This indicates that our control sample is representative of community controls.[4]

The first-episode AN participants included in the present study did not show an association between higher olfactory sensitivity and less severe AN symptoms (lower body dissatisfaction, and higher BMI), previously demonstrated in adults with AN.[64] However, differences of sample characteristics render direct comparisons difficult. Moreover, the clinical homogeneity of the present sample with first-episode AN may have reduced the variability of symptom severity, thus making it difficult to identify associations. Furthermore, olfactory sensitivity in our participants was not related to self-reported symptoms of depression or anxiety. Finally, excluding participants with depressive or anxiety disorders, participants who smoke, or participants undergoing SSRI treatment, did not change the findings regarding olfactory sensitivity. Thus, our exploratory analyses suggest that differences in olfactory sensitivity between controls and the clinical groups were not affected by the presence of psychiatric comorbidity although depression and anxiety as primary disorders are associated with reduced and increased olfaction, respectively.[21,22] We speculate that the associations between these disorders and olfactory sensitivity may be differentially modified by the presence of AN. Hence, AN may “overrule” possible influences of depression on the capacity of olfaction. Furthermore, even though first-episode AN as well as anxiety are associated with superior olfaction ability, our results suggest there may not be an additive effect, when both disorders are present. However, the limited size of subgroups with these comorbid disorders in our study did not allow for formal subgroup analyses.

Regarding odor identification, lack of controlling for comorbid depressive disorder may partly explain the divergent findings in prior studies[11] that report either reduced identification[19,60,61] or no significant alterations compared with controls.[15,20,21,64,65] In the current study, the subgroup of participants with first-episode AN without a depressive disorder displayed more precise odor identification than controls, which is in line with an earlier observation in adolescents with AN without comorbidity.[66] However, the dimensional measure of depression symptoms, BDI-Y, did not significantly influence the between-group differences in odor identification. Possibly, the BDI-Y items may represent more transient aspects of emotional distress that frequently accompany a diagnosis of AN than a manifest diagnosis of a depressive disorder.[67]

The presence of anxiety may improve odor identification in individuals with first-episode AN, because symptom provoking stimuli, such as food, activate brain regions associated with anxiety, e.g. hippocampus and amygdala, in individuals with AN.[68] Indeed, nine of 16 target smells in the Odor Identification Test represent a smell from foods or beverages. A higher level of anxiety is significantly associated with enhanced odor identification in individuals without AN,[22,69] and anxiety is associated with a tendency to attend to threatening information.[70] Superior odor identification in individuals with AN may therefore represent an attentional bias toward feared stimuli related to eating.[16]

Our findings further suggest that less accurate odor identification is associated with higher levels of body dissatisfaction in participants with first-episode AN. This points to a more general impairment of interoceptive awareness,[7] which is common in individuals with AN and reflected in their distorted body image and body dissatisfaction.[71] Altered interoceptive awareness may promote aversive conditioning of the bodily cues of hunger and satiety in AN and thus lead to a top-down cognitive avoidance of food-related stimuli such as smells.[7] We thus hypothesize that altered interoceptive awareness may explain the association between less accurate odor identification and higher levels of body dissatisfaction.

In summary, odor identification may be superior in the initial phases of AN, as confirmed on the group level in our study, but this effect may be counteracted by a top-down cognitive avoidance based on altered interoceptive awareness. Furthermore, a longer duration of illness may impede odor identification, in line with decreasing olfactory sensitivity in adults with a chronic course of the disorder.[19,66] This was confirmed in a sample of adults with AN, in which only participants with the lowest weight, associated with length of illness, displayed reduced odor identification.[19] Thus, we have reason to speculate that avoidance related to altered interoceptive awareness, or factors related to duration of starvation, or both, may counteract the otherwise heightened odor identification in individuals with first-episode AN.

Participants with first-episode AN indicated less importance of olfaction in everyday life than controls, whereas participants recovered from AN indicated a similar level of importance of olfaction as the controls. The difference was only statistically significant when food-related items were included and may point to the importance of food-related content. Although highly speculative, we suggest that not ascribing subjective importance to olfaction in first-episode AN may reflect aspects of the above-mentioned top-down avoidance following attentional bias toward feared, food-related stimuli in acutely ill AN individuals, although on the group level they display heightened odor identification.

Due to the cross-sectional design of the present study, it is impossible to know whether the observed higher olfactory sensitivity and more accurate olfactory identification in participants with first-episode AN were present before the onset of AN, as well as how those abilities will develop further after this early phase of AN. Although we cannot entirely exclude the possibility that the higher olfactory sensitivity in recovered participants is a cohort effect, our findings suggest that higher olfactory sensitivity in AN may be relatively independent of weight loss and other characteristics of the acute phase, whereas the finding of more accurate odor identification is only present in the initial phase of AN.

### Strengths and limitations

The major strength of the current study is the detailed characterization of the clinical groups and the inclusion of participants recovered from AN. Moreover, participants with first-episode AN had a short duration of illness thereby avoiding the potential confounding effects of chronicity, and the recovered participants met strict and multifaceted criteria for recovery. However, there were also some limitations. First, our findings cannot be generalized to males with AN, as sex differences in olfaction have been noted.[5] Second, we did not employ the new and revised algorithm of ADOS module 4[72] as an outcome measure of social function. Third, the differences in administration of the Sniffin’ Sticks Test may influence direct comparisons with previous studies that have employed this test. The reliability of odor threshold determination is lower than other olfaction measures and has been questioned for several reasons, including the use of only one target odor.[22] Furthermore, we used the standard *n*-buthanol as target odor for the Sniffin´ Sticks Odor Threshold Test. *N*-buthanol stimulates not only the olfactory system but also the trigeminal system of nasal receptors,[73] and therefore the observed sensitivity may not be an exclusive effect of the olfactory system. Lastly, we did not control for state of satiety in participants when testing olfaction. Future studies may benefit and possibly obtain more consistent results by including state of satiety in participants.

## Conclusion and Applications

We observed enhanced olfactory sensitivity in participants with first-episode AN and in recovered participants, whereas only participants with first-episode AN and without comorbid depression displayed superior odor identification. Less accurate odor identification was associated with higher levels of body dissatisfaction in participants with first-episode AN, which ascribed less importance to olfaction than did the other two groups. We observed no association between olfaction and social function in these participants.

Olfaction has been proposed as a biomarker for diagnosis and prognosis in a range of disorders.[22] However, given the inconsistency of findings, populations and methodologies in studies of olfaction in AN to date, [11] its use as a marker for diagnosis seems unwarranted. Moreover, the theoretical framework that places olfaction within a generally disturbed processing of sensory perceptions in AN via anxiety and avoidance, needs further investigation.[7,35] If confirmed, olfaction might be a more distal and hence less fear-provoking area to begin habituation and reversal training than i.e. the sense of satiety. To the degree that the heightened olfactory sensitivity in AN is aversive, clinical care programs may take this evidence into account by accommodating food and environments without strong smells, thus making the process of re-nourishment less stressful.

## Supporting Information

S1 Dataset The data used for conclusions in the article can be found in the S1 Dataset. (XLS)
